# Anatomical and Microscopical Observations on the Structure of the Nervous System

**Published:** 1839-04

**Authors:** 


### ART. X.

*Observationes Anatomicæ et Microscopicæ de Systematis Nervosi Structura. Auctore* ROBERTO REMAK, Med. et Chir. Dr.

*Anatomical and Microscopical Observations on the Structure of the Nervous System.* By ROBERT REMAK, M.D. *With two copper-plates.* —*Berlin*, 1838. 4to. pp. 41.

THIS memoir not having come to hand when the article on the structure of the brain and nerves in our Twelfth Number was printed, we now give an abstract of it by way of supplement.

The memoir is a thesis which the author wrote on taking his medical degree at the University of Berlin. It is divided into two sections, treating, respectively, of the peripheral parts of the nervous system, and of the central parts. The plates are very expressive and well executed, from the author's own drawings.

1. *Primitive Tubules and Fibres of the Cerebro-spinal Nerves.* As mentioned in the article in our former Number, Remak thinks he has determined, "that the contents of the primitive tubules, both of the nerves and of the central parts, is by no means an oily mass, or one composed of globules, or an altogether amorphous mass, but a *fibre* which is solid, plane, extremely pellucid, having straight parallel margins, an even but somewhat rough surface, and a remarkably strong texture, so that it is less easily torn than its sheath."

2. *Organic Fibres of the Sympathetic Nerve and of the Cerebro-spinal Nerves.* In our former article, p. 412, we said, that it is a question whether the sympathetic really contain any fibres *sui generis.* Retzius and Müller observed, in certain nerves, gray filaments from the sympathetic, which did not immediately intermix with the white fibres, but preserved their colour and situation for a considerable length. Valentin, as we saw, attributes the colour not to gray filaments but to the pressure of the nucleated globules, which he and Purkinje discovered in the gray substance of the brain and spinal marrow, and in the ganglions. According to Remak's observations, however, "the peculiar colour and constitution of the sympathetic nerve and its branches are not produced by an intermixture of nucleated globules, but by the peculiar structure of primitive fibres originating from the ganglions:" these primitive fibres (as they are not tubular, that is, not enveloped in any sheath, but are naked,) are extremely pellucid as if gelatinous, much more slender than the generality of primitive tubules, and almost always presenting longitudinal lines on the surface. They readily resolve themselves into very delicate threads; they frequently present oval nodules in their course, and are more or less abundantly covered by small corpuscles, which are oval or round, seldom irregular, simply or compoundly nucleated, and, as to size, almost equalling the *nuclei of the nucleated globules.* These fibres, Remak calls *organic fibres.* They must not be confounded with the *primitive tubules* of the brain and nerves, nor with the *primitive fibres* which these tubules, according to Remak, contain. Remak says, these organic fibres are easily observed in all the cerebro-spinal nerves, and at every part of their course, by means of the microscope. He has found them, though in small quantity, both in the anterior and posterior roots of the spinal nerves.

Gray fibres of the sympathetic, or organic fibres, then, are found in the cerebro-spinal nerves. Remak has found, on the other hand, "white nervous threads from the roots of the spinal nerves joining the sympathetic, and running separately among its gray parts for very long distances." "It often happens," he continues, "that in the gray cords of animals recently killed, white threads may be followed as far as the ganglions, where the primitive tubules, after they have formed plexuses, mingle in greater or smaller quantity with the various branches proceeding from the ganglions."

It thus happens that the cords arising from the ganglions of the sympathetic vary much in colour and constitution. Many are merely gray, almost gelatinous, opaque, and composed for the most part of *organic fibres;* though they almost always present, on one or other side, very slender white threads, discernible by the naked eye or by the assistance of a simple lens, composed of *primitive tubules.* Other cords again, especially in the thoracic and abdominal part of the sympathetic, incline to a *white colour,* and contain a greater quantity of *primitive tubules* than the merely gray cords.

3. *Ganglions of the Sympathetic and Spinal Nerves.* In regard to Valentin's account of the structure of the ganglions, Remak remarks: "but the point, without doubt, of the greatest importance for the understanding of the nature of the ganglions, Valentin did not nor could know; *for the organic fibres arise from the very substance of the nucleated globules.*" Remak is of opinion that, as the organic fibres, which constitute the greater part of the sympathetic nerves, take their origin from those nucleated globules, by the aggregation of which the ganglions are especially formed—the sympathetic ganglions must he considered as *true centres of the organic nervous system.* He is further of opinion that, as the differences between the sympathetic and the spinal ganglions consists only in the quantity of fibres thence arising, and in the number of nuclei, *the spinal ganglions themselves appear to belong to the organic nervous system.*

4. *Spinal marrow.* *a.* Anatomical observations. Rolando makes mention of a peculiar substance, which he calls *gelatinous* in the posterior part of the spinal marrow. Remak has made some further observations on this subject. According to him, it covers not only the posterior margins of the posterior cornua of the gray substance of the spinal marrow, but a very delicate lamina of it is also continued at the internal margins of these cornua, in such a way, that between the posterior white commissure (that is, the continuation of the white cortex of the spinal marrow from the one side to the other, at the bottom of the posterior fissure), and the commissure between the two lateral portions of the central gray substance, there is a commissure of the gelatinous substance under consideration. Hence, Remak counts four commissures, by which the lateral parts of the spinal marrow are joined, viz., a posterior white commissure; a gelatinous commissure; a gray or spongy commissure; and an anterior white commissure.

On each side of the apex of the calamus scriptorius there are grayish tubercles, the *tubercula cinerea* of Rolando. These, Remak has found to be a true continuation of the gelatinous substance of the spinal marrow, of which they exhibit the same characters as regards colour, consistence, and internal structure. The gelatinous tubercles are joined by a commissure, the continuation of the gelatinous commissure of the spinal marrow; and behind this, Remak has also found a continuation of the posterior white commissure, joining the posterior pyramids in such a way that, together with the gelatinous commissure at that place, there is formed a sort of transverse frenulum over the entrance of the short canal described by Rosenthal at the apex of the calamus scriptorius.

According to Burdach, the filament which forms the inferior extremity of the spinal marrow contains gray substance. Remak's most recent observations go to show that the matter which is contained in the filament, or rather in that slender canal, is *gelatinous substance.*

*b.* Microscopical observations. In the gray or spongy substance of the spinal marrow, Valentin observed nucleated globules, but here also he overlooked the most important point; for from the nucleated globules of the gray substance, which generally exceed in size those of the ganglions and of the cerebrum, microscopical fasciculi also arise, equalling in breadth nearly the primitive tubules. These microscopical fasciculi consist of several non-tubular but generally rough fibres, often disposed, as it were, tortuously. They are sometimes not unlike those which arise from the globules of the ganglions, if the nodules and nucleated corpuscles which occur in the organic fibres be kept out of view; sometimes again they resemble the primitive fibres (of the tubules), from which, however, they differ by their greater roughness and greater tendency to ramification.

In the gelatinous substance of the spinal marrow, Remak found corpuscles for the most part oval, but sometimes round, always a little flattened and pellucid; sometimes, especially in the ox, of a bright yellowish red colour, and containing near the surface a small nucleus. As concerns structure and magnitude, they resemble more or less the *nuclei of the nucleated globules.* They are remarkably like the greater part of the nucleated corpuscles found contained in the organic fibres; often also, especially when they are coloured, like the corpuscles of the blood of the triton. The corpuscles, under consideration, although very often observed lying free, yet appear to be joined intimately with that texture which, in addition to these, composes the gelatinous substance. On account of the remarkable softness and tenuity of this texture, Remak has succeeded in recognizing the disposition of the fibres of which it consists, only in some places of the gelatinous substance to be mentioned below. But the very delicate primitive tubules which are observed in the gelatinous substance are those decussated in a manifold way; they appear, however, to pass through it only, not in any way to terminate in it.

The inferior extremity of the spinal marrow, as it is composed entirely of gelatinous substance, contains in general all the parts just described; but on account of several differences which it presents, it requires a particular description. The nucleated corpuscles, which increase in quantity so much towards the lower end, that the lowest part of the terminal filament appears to consist of nothing but corpuscles of this kind, begin in the cylindrical part to differ from those of the rest of the gelatinous substance, inasmuch as they contain much more frequently two or three or more small nuclei, and as there are intermixed among them very many corpuscles of almost the same form and size, but as yet more pellucid, quite plane, and wanting nucleoli. But as to the structure of the fibres, which, in addition to the nucleated corpuscles, compose the gelatinous substance, Remak could only observe this much, that at the very extremity of the central filament in the sheep, they formed a very delicate network, abundantly covered by these different corpuscles.

Lastly, the lateral branches of the terminating filament of the spinal marrow, which have a very great external resemblance to the sympathetic nerves, are so constructed internally, that, with the exception of the greater tenuity and delicacy of the parts, it has not happened to Remak to observe any more important difference from the organic nerves. They consist, for the greater part, of very slender naked fibres, in the course of which are frequently found nucleated corpuscles, varying as to form and structure, in the same way as in the organic nerves and lower end of the spinal marrow, and of more delicate primitive tubules which generally appear to correspond, as to external constitution, in a remarkable manner with the tubules of the organic nerves.

Remak has not been able to make out anything certain regarding the relation between the roots of the spinal nerves and the different substances composing the spinal marrow. He says, " one thing I am persuaded of is, that the fibres of the roots of the nerves have not such a simple origin as that of passing immediately into the longitudinal fibres of the spinal marrow." He adds, in a note, that he has seen fibres from the posterior roots perforating horizontally the white substance in the interstice between the anterior and posterior roots, and running to the external part of the posterior cornua of the gray substance. The connexion between the roots of the spinal nerves and the gray substance of the spinal marrow, observed first by Bellingeri, and mentioned again by Weber, (Hildebrandt's Anatomie, Bd. iii. p. 374,) has been more recently insisted on by Mr. Grainger.*

5. *Brain and Cerebellum.* In the article in our Twelfth Number, p. 413, we spoke of certain caudate nucleated globules, observed by Purkinje in the yellow substance of the cerebellum, by Valentin in the yellow substance of the brain, and by Müller in the medulla oblongata of cyclostomatous fishes. According to Remak's observations, all the varieties of globules described by these investigators depend merely on this, that from each nucleated globule of the cerebellum, both in the yellow and in the gray substance, as also in the spinal marrow, in the cerebrum and in the ganglions, one or more microscopical funiculi, composed of more delicate fibres arise, differing in no important particular from those observed in the spinal marrow.

Remak never could observe the terminating loops of the primitive tubules situated between the white and gray substances of the brain and cerebellum, as described by Valentin, in such a way as to be able to conclude that these loops should be considered the true terminations of the tubules in the encephalon. It seemed to him rather that the primitive tubules run tortuously, much in the same way as in the exterior parts of the ganglions and in the spongy substance of the spinal marrow, bending and rebending several times, as they gradually approach the surface of the cerebrum. Whence it is, that frequently there are observed arches of tubules, the crura of which are directed toward the very surface of the brain.

Remak has not determined anything in regard to the peripheral terminations of the nerves; he seems satisfied of the correctness of Schwann's observations regarding the microscopical networks of the cutaneous nerves.

In dissecting brains, of the larger mammalia especially, there is easily observed a purely gray, almost gelatinous, stratum, seldom exceeding half

* See the notice of Mr. Grainger's Essay on the Structure and Functions of the Spinal Cord, in our Volume V. p. 486.

a line in thickness, except in one place to be described below. This stratum is more or less separated by a white interstice of almost the same thickness, from the cortical substance, but in regard to its course it is almost everywhere parallel with the gray substance. This matter noticed only, and that but slightly, by Gennari and Soemmering, is composed, according to Remak's observations, for the greater part, like the gelatinous substance of the spinal marrow, of nucleated corpuscles and very slender non-tubular fibres. This gelatinous substance of the brain descends into the pes Hippocampi, and there swelling, forms a sort of ganglion several lines in thickness. The intumescence constitutes, as it were, the nucleus of the pes Hippocampi, and is separated by a thin white layer from the cortical substance of that gyrus, from the evolution of which the pes Hippocampi is in part produced.

Remak concludes the descriptive part of his very interesting paper by the following curious observation. He has found that the *acervulus*, which is met with in the pineal gland and in the plexuses of the pia mater, consists of globules equalling or exceeding the largest nucleated globules. If the globules of the acervulus be subjected to the action of muriatic acid, carbonic acid is evolved, and there are left pellucid globules, containing a reddish nucleus, and a nucleolus like the nucleated globules of the ganglions.

---

